# Social Engagement is associated with better cognition in Quilombola Communities: Insights for Engaging underserved Brazilian Populations in Alzheimer’s Disease Research

**DOI:** 10.1002/alz.090989

**Published:** 2025-01-09

**Authors:** Sharon Sanz Simon, Carolina Cappi, João de Deus Cabral Junior, Gilberto Sousa Alves, Bruno Luciano Carneiro Alves de Oliveira

**Affiliations:** ^1^ Taub Institute for Research in Alzheimer’s Disease and the Aging Brain, Columbia University, New York, NY USA; ^2^ Old Age Research Group (PROTER), Medical School, University of São Paulo, São Paulo Brazil; ^3^ Cognitive Neuroscience Division, Columbia University, New York, NY USA; ^4^ Department of Psychiatry, Icahn School of Medicine at Mount Sinai, New York, NY USA; ^5^ Federal University of Maranhão, Pinheiro, MA Brazil; ^6^ Translational Psychiatry Research Group, Faculty of Medicine, Federal University of Maranhão, São Luís, MA Brazil; ^7^ Federal University of Maranhão, São Luis, MA Brazil

## Abstract

**Background:**

Quilombos are settlements founded by descendants of runaway slaves in Brazil, typically located in remote rural areas. Quilombola communities typically present poor basic sanitation and health, high levels of illiteracy, and limited access to social and health services, especially among older adults. Therefore, the Quilombola population is socially vulnerable and likely to present an increased risk for dementia, although underrepresented in aging/dementia research. Future research is needed to map dementia risk factors in this population. The present study aims to investigate the association between cognition and socio‐cultural engagement, potentially protective for dementia.

**Method:**

This study is part of the Population Survey of the Living Conditions and Health Status of Older Persons Living in Quilombola Communities in the Baixada Maranhense (IQUIBEq) project, a cross‐sectional household survey conducted in 11 Quilombola communities in Maranhão (Brazil). The study comprised 221 older adults (60‐104 years), selected in partnership with local social services and community health workers. Participants completed a health survey and a cognitive screening (Mini‐Mental State Examination). Demographics, cardiovascular risk factors (waist‐to‐hip ratio and BMI), cognitive performance, and social engagement were described. Regression analysis examined the association between social engagement and cognition. Our models controlled for age, sex, race, education, family income, and cardiovascular risk factors. We also examined if sex and age moderated the association observed.

**Result:**

Higher social engagement is associated with better cognition (p <.001) above and beyond demographics and cardiovascular risk factors. We observed the social activities that mostly drove our results were attending community meetings (e.g., community movements or resident associations) and attending religious/faith services. Moreover, age and sex did not moderate the association between social engagement and cognition.

**Conclusion:**

Social engagement may be a key aspect of cognitive/brain health in the Quilombola population, a group that faces poor life conditions. Social engagement may not only provide social/emotional support among the community members but can facilitate access to basic needs (e.g., housing, sanitation). Other potential mechanisms are the cognitive stimulation present in social interactions and the effects of spiritual/faith/religious engagement. Although the results are limited by the cross‐sectional design and survival bias, they may inform public health strategies in underserved populations.

